# Re-evaluating classical body type theories: genetic correlation between psychiatric disorders and body mass index

**DOI:** 10.1017/S0033291718000685

**Published:** 2018-04-13

**Authors:** Masashi Ikeda, Satoshi Tanaka, Takeo Saito, Norio Ozaki, Yoichiro Kamatani, Nakao Iwata

**Affiliations:** 1 Department of Psychiatry, Fujita Health University School of Medicine, Toyoake, Aichi, Japan; 2 Department of Psychiatry, Nagoya University Hospital, Nagoya, Aichi, Japan; 3 Department of Psychiatry, Nagoya University, Graduate School of Medicine, Nagoya, Aichi, Japan; 4 Laboratory for Statistical Analysis, RIKEN Center for Integrative Medical Sciences, Yokohama, Japan; 5 Center for Genomic Medicine, Kyoto University Graduate School of Medicine, Kyoto, Japan

## Introduction

Body type theories, proposed by Kretschmer and Sheldon, are historical concepts that attempt to correlate somatotypes and personalities or psychiatric disorders (Kretschmer, 1925; Sheldon, 1940). Accordingly, Kretschmer classified four types of people: (1) the asthenic type who has a slender body (‘leptosome’) and is more prone to schizophrenia (SCZ); (2) the pyknic type who has a round body and is likely to become manic-depressive illness [bipolar disorder (BD)]; (3) the athletic type with a muscular body who may suffer from epilepsy; and (4) the dysplastic type who cannot be classified as any of the other three types (Kretschmer, 1925). Sheldon had a similar insight, classifying people into ectomorphic (corresponding to Kretschmer's asthenic type), endomorphic (corresponding to the pyknic type), and mesomorphic (corresponding to the athletic type). Interestingly, Sheldon also claimed that there was a deeper connection, with genetic components being linked to both the somatotypes and personality (Sheldon, 1940). Such classifications proposed by these celebrated psychiatrists or psychologists were based on their observational surveys in the beginning of the twentieth century; however, scientific evidence thereof is limited (Sorensen *et al.* 2006; Zammit *et al.* 2007).

Nevertheless, recent genetic studies have re-evaluated their body type theories by conducting genetic correlation analysis, which employs genetic association results from genome-wide association studies (GWASs) to examine the genetic relationship between two independent phenotypes, that is, theoretically, excluding environmental factors. The first study reported a significant correlation between SCZ and low body mass index (BMI) in a European (EUR) ancestry (Bulik-Sullivan *et al.* 2015); however, no correlation was observed between BD and BMI (Bulik-Sullivan *et al.* 2015). Following this, we conducted a study in which we replicated a significant negative correlation between SCZ and BMI, but again found no correlation between BMI and BD in the Japanese samples (Akiyama *et al.* 2017). More recently, the PGC2 major depressive disorder (MDD) group has reported a positive correlation between BMI and MDD (Major Depressive Disorder Working Group of the PGC, 2017), using EUR samples.

## Method

These results motivated us to conduct a meta-analysis (using the ‘metafor’ package in R project: https://www.r-project.org/) to estimate the combined polygenic relationships between two datasets from different populations:

(1) for SCZ and BD in EUR datasets, we updated the genetic correlation using LD score regression analysis (Bulik-Sullivan *et al.* 2015) (removing MHC SNPs from 26Mb to 34Mb and using SNPs with imputation INFO >0.7) based on the Psychiatric GWAS Consortium data only for the EUR [PGC2(49) for SCZ (Schizophrenia Working Group of the Psychiatric Genomics Consortium, 2014) and PGC-BD (Psychiatric GWAS Consortium Bipolar Disorder Working Group, 2011: https://www.med.unc.edu/pgc/results-and-downloads/] and GIANT GWAS 2015 (Locke *et al.* 2015: https://portals.broadinstitute.org/collaboration/giant/images/1/15/SNP_gwas_mc_merge_nogc.tbl.uniq.gz).
(2) For the Japanese SCZ samples, we used our published results (Akiyama *et al.* 2017). For the Japanese BD subjects, we updated the genetic correlation between the BMI (Akiyama *et al.* 2017) and the BD results (INFO >0.7), which we also published recently (Ikeda *et al.* 2018).
(3) In the MDD analyses, we used the results of the PGC2-MDD (Major Depressive Disorder Working Group of the PGC, 2017) in a EUR dataset [BMI dataset was GIANT GWAS 2015 (Locke *et al.* 2015)], and
(4) calculated genetic correlations using the CONVERGE Consortium [Chinese females only: INFO >0.816 because summary statistics over this threshold were publicly available: (CONVERGE consortium, 2015)] and our BMI results [Japanese females only: INFO >0.816: (Akiyama *et al.* 2017)] for the dataset for East Asian ancestry (EAS).

As an exploratory investigation, and in an attempt to replicate the findings from the current meta-analysis, we also conducted trans-ethnic genetic correlation analysis (BMI in EUR and psychiatric disorders in EAS, and *vice versa*) using the Popcorn software (Brown *et al.* 2016), following a default protocol (https://github.com/brielin/Popcorn).

All procedures contributing to this work complied with the ethical standards of the relevant national and institutional committees on human experimentation and with the Helsinki Declaration of 1975, as revised in 2008.

## Results

**Fig. 1.**
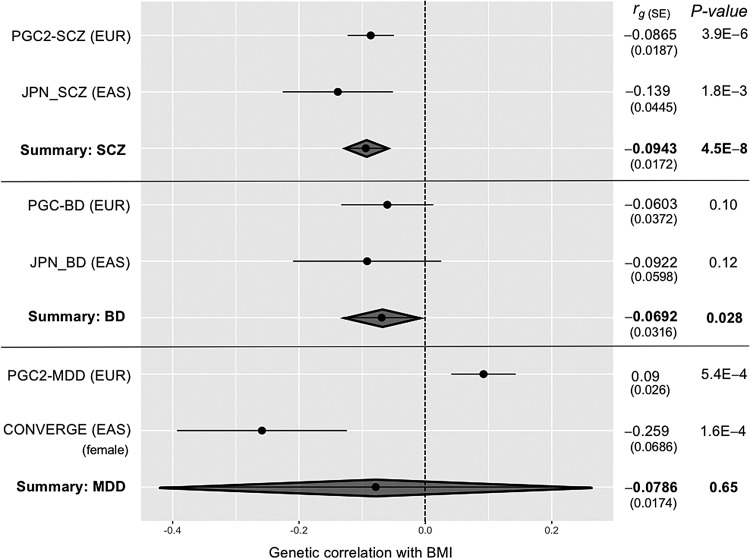
Meta-analysis of the genetic correlations (*r*_*g*_) between body mass index (BMI) and schizophrenia/bipolar disorder/major depressive disorder (SCZ/BD/MDD). s.e.: standard error. No heterogeneity was observed either in SCZ/BD analyses (*p* = 0.28 and *p* = 0.65 for SCZ and BD, respectively) but significant heterogeneity was found in MDD analyses (*p* = 2.0 × 10^−6^). Therefore, random-effect model was applied in the meta-analysis for MDD. PGC2-SCZ (EUR): SCZ genome-wide association study (GWAS) in the European (EUR) ancestry (33 640 SCZ *v.* 43 456 controls: Schizophrenia Working Group of the Psychiatric Genomics Consortium, 2014). BMI was calculated on the basis of maximum 322 154 subjects in the European ancestry (Locke *et al.* 2015). JPN-SCZ: SCZ GWAS in the Japanese ancestry (1987 SCZ *v.* 9788 controls: Akiyama *et al.* 2017). BMI was calculated on the basis of 158 284 subjects in the Japanese ancestry (Akiyama *et al.* 2017). PGC-BD: BD GWAS in the European ancestry (7481 BD *v.* 9250 controls: Psychiatric GWAS Consortium Bipolar Disorder Working Group, 2011). BMI was calculated on the basis of maximum 322 154 subjects in the European ancestry (Locke *et al.* 2015). JPN-BD: BD GWAS in the Japanese ancestry (2964 BD *v.* 61 887 controls: Ikeda *et al.* 2018). BMI was calculated on the basis of 158 284 subjects in the Japanese ancestry (Akiyama *et al.* 2017). PGC2-MDD: MDD GWAS in the European ancestry (130 664 MDD *v.* 330 470 controls: Major Depressive Disorder Working Group of the PGC, 2017). BMI was calculated on the basis of maximum 322 154 subjects in the European ancestry (Locke *et al.* 2015). CONVERGE: MDD GWAS in the East Asian (EAS) ancestry (females only: 5303 MDD *v.* 5337 controls: CONVERGE consortium 2015). BMI was calculated on the basis of 72 390 female subjects with Japanese ancestry (Akiyama *et al.* 2017).

In Fig. 1, the forest plots for the genetic correlations between BMI and SCZ/BD/MDD are shown. The combined estimate of *r*_*g*_ for SCZ was −0.094 [fixed effect model: standard error (s.e.) = 0.017, *p* = 4.5 × 10^−8^], and for BD, there was a marginally significant correlation at −0.069 (fixed effect model: s.e. = 0.032, *p* = 0.028) with no statistical heterogeneities (*p* > 0.05). Interestingly, the meta-analysis for MDD showed significant heterogeneity (*p* = 2.0 × 10^−6^), in the opposite direction of the correlation between the EUR (i.e. positive correlation) and EAS (i.e. negative correlation) samples.

The exploratory trans-ethnic genetic correlation analysis partially supported the findings from the meta-analysis between SCZ/BD and BMI: negative correlation between BMI defined by EUR and SCZ defined by EAS (and *vice versa*; see online Supplementary Table S1). For BD, we replicated the finding of the meta-analysis in our examination of [BMI (from EUR)/BD (from EAS)], but in the reverse analysis [BMI (from EAS)/BD (from EUR)], we observed non-significant trends between BMI and BD (e.g. *ρ*~−0.04, *p* value for ‘non-correlation test’ ~0.10; see online Supplementary Table S1). However, it is difficult to interpret these results, since (1) the sample sizes for BMI GWAS differed substantially (the EUR sample was twice the size of EAS) and (2) this analysis aimed at examining not only trans-ethnic but trans-phenotype factors as well.

## Discussion

The results of the polygenic correlation between low BMI and SCZ, which was measured or diagnosed by modern practice, provide some support for the body type theories by Kretschmer and Sheldon, although as noted previously, they proposed the theory on the basis of their observation of people or diagnosis in their era. Therefore, this result for SCZ sheds light on Kretschmer and Sheldon's insight, which proposed diagnostic categories for psychiatric disorders based on the somatology; it is surprising that their classical and well-known hypothesis could be explained by genetic factors, although Kaplan and Sadock's textbook, for example, regarded Kretschmer's achievement as a ‘history’ (Sadock *et al.* 2014). Nevertheless, our genetic correlation analysis did not correspond to the ‘body type’ analysis for BD, in which Kretschmer and Sheldon assumed the tendency of the round body in BD patients; rather, the correlation was in the opposite direction (i.e. low BMI was associated with BD), despite the relatively low statistical significance of both the meta-analysis and the trans-ethnic/phenotype analysis.

In addition, from a clinical perspective, the current genetic results may indicate that patients with SCZ (and possibly BD) tend to have lower BMI presumably *before* the ‘onset’ (i.e. influenced by fewer confounding ‘environmental factors’). In a real clinical setting, the major and defining problem of the patients with SCZ and BD is obesity (Manu *et al.* 2015; Strassnig *et al.* 2017). Therefore, we speculate, on the basis of our current results, that obesity in SCZ or BD may not be attributable entirely to genetic factors but might be greatly influenced by several environmental factors, such as medications (e.g. antipsychotics), lifestyle, and living conditions of modern society, especially during the prodromal period and after the ‘onset’ (Correll *et al.* 2014) or gene–environment correlation/interaction.

The results based on MDD are also of interest. The high level of heterogeneity between BMI and MDD in each population might reflect the different clinical backgrounds of the MDD samples (PGC2 for EUR and CONVERGE for EAS). For MDD samples from EUR (correlated with high BMI), the proportion of MDD individuals with atypical features, characterized by increased appetite and body weight during the depressive phase, was modest. In this context, a recent subgroup study targeting the same PGC2 sample found that MDD with atypical features was correlated with high BMI (*r*_*g*_ = 0.53, *p* = 6.3 × 10^−4^). In contrast, more typical features of MDD (decreased appetite and body weight, probably including melancholia) exhibited a nonsignificant trend of negative correlation with BMI (*r*_*g*_ =  −0.28, *p* = 0.06; Milaneschi *et al.* 2017). Therefore, it could be inferred that the evidence of a shared genetic component between MDD with atypical features and high BMI is attributable to the significant positive correlation between overall MDD and BMI in EUR datasets (Major Depressive Disorder Working Group of the PGC, 2017). On the contrary, for MDD samples from EAS (CONVERGE consortium, 2015), the primary feature related to phenotyping is the predominance of melancholia (about 85%), which is generally associated with low appetite and body weight loss during the depressive course; the result in our study, a negative correlation between BMI and MDD in EAS, is presumably compatible with the sample proportion of CONVERGE. Nevertheless, there were a few limitations in the interpretation of the results; first, our results for genetic correlation in EAS are based solely on female subjects because the CONVERGE consortium targeted female MDD to select a purer phenotype (CONVERGE consortium, 2015). Second, it should also be stressed that the comparison of MDD in the EAS was not perfectly matched in terms of ethnicity; there were MDD subjects with Chinese and BMI subjects having Japanese ancestry.

In conclusion, a trans-ancestry meta-analysis of the genetic correlation between psychiatric disorders and BMI indicated that the negative correlation with SCZ supported classical body type theories proposed in the last century, but found a negative correlation between BD and BMI, opposite to what would have been predicted. In addition, our results indicate that clinical subtypes of MDD play a role in the genetic correlations with BMI; however, further study is required to achieve conclusive results. Genetic correlation analysis is clearly a powerful tool for (re)discovering unexpected connections between psychiatric disorders and other phenotypes (Bulik-Sullivan *et al.* 2015). Therefore, the current trend toward conducting such analyses intensively could detect new links or reinforce previously identified ones, thereby helping us to better understand psychiatric disorders.

## Supplementary material

For supplementary material accompanying this paper visit https://doi.org/10.1017/S0033291718000685. click here to view supplementary material
