# Supplementary material for: Re-evaluating classical body type theories: genetic correlation between psychiatric disorders and body mass index
Source: Psychol Med. 2018 Apr 13;48(10):1745–8. doi: 10.1017/S0033291718000685 (PMC6088781; doi:10.1017/S0033291718000685)
Supplement: Supplementary file 1 [file S0033291718000685sup001.docx]

|  |  |  |  |  |  |  |  |  |  |  |
| --- | --- | --- | --- | --- | --- | --- | --- | --- | --- | --- |
| EUR samples | EAS samples | ρge | SE | P for \|ρge\|>0* | P for \|ρge\|<1** |  | ρgi | SE | P for \|ρgi\|>0* | P for \|ρgi\|<1** |
|  | SCZ (Akiyama et al.) | -0.191 | 0.047 | **2.3.E-05** | 0 |  | -0.187 | 0.043 | **6.6.E-06** | 0 |
| BMI (Locke et al.) | BD (Ikeda et al.) | -0.137 | 0.052 | **0.0040** | 0 |  | -0.133 | 0.048 | **0.0030** | 0 |
|  | MDD (CONVERGE) | -0.348 | 0.062 | **1.1.E-08** | 0 |  | -0.338 | 0.063 | **4.8.E-08** | 0 |
| SCZ (PGC2) |  | -0.050 | 0.023 | **0.0147** | 0 |  | -0.043 | 0.022 | **0.0250** | 0 |
| BD (PGC) | BMI (Akiyama et al.) | -0.043 | 0.034 | 0.1023 | 0 |  | -0.041 | 0.032 | 0.1018 | 0 |
| MDD(PGC2) |  | NA | NA | NA | NA |  | NA | NA | NA | NA |
|  |  |  |  |  |  |  |  |  |  |  |

**Supplementary Table S1: Trans-ethnic genetic correlation analysis (BMI and psychiatric disorders)**

ρge: genetic effect correlation (the correlation coefficient of the population-specific allele-variance-normalized SNP effect sizes), ρgi: genetic impact correlation (the correlation coefficient of the per-allele SNP effect sizes)

SCZ: schizophrenia, BD: bipolar disorder, MDD: Major depressive disorder, SE: standard error, NA: not analyzed due to unavailability of whole genome summary statistics

*test that the genetic correlation is greater than 0 (one-sided)

** test that the genetic correlation is less than 1.0
